# Optic nerve and chiasm hemangioblastomas in von Hippel-Lindau disease: report of 12 cases and review of the literature

**DOI:** 10.3389/fonc.2024.1334564

**Published:** 2024-07-09

**Authors:** Evelynn Vergauwen, Jan-Helge Klingler, Marie T. Krüger, Christine Steiert, Robert Kuijpers, Steffen Rosahl, Anne-Marie Vanbinst, Corina Emilia Andreescu, Sven Gläsker

**Affiliations:** ^1^ Department of Neurosurgery, Vrije Universiteit Brussel, Jette, Belgium; ^2^ Department of Neurology, Algemeen Ziekenhuis (AZ) Klina, Brasschaat, Belgium; ^3^ Department of Neurosurgery, Freiburg University Medical Center, Freiburg, Germany; ^4^ University College London (UCL) Functional Neurosurgery Unit, National Hospital for Neurology and Neurosurgery, London, United Kingdom; ^5^ Department of Ophthalmology, Vrije Universiteit Brussel, Jette, Belgium; ^6^ Department of Neurosurgery, Helios Klinikum Erfurt, Erfurt, Germany; ^7^ Department of Radiology, Universitair Ziekenhuis Brussel, Jette, Belgium; ^8^ Department of Endocrinology, Universitair Ziekenhuis Brussel, Jette, Belgium; ^9^ Neurosurgery Section, Gesundheitsverbund Landkreis Konstanz (GLKN), Singen am Hohentwiel, Germany

**Keywords:** von Hippel-Lindau, eye, optic nerve, optic chiasm, optic hemangioblastoma, hemangioblastoma, review, VHL

## Abstract

**Introduction:**

Optic nerve and chiasm hemangioblastomas are rare tumors, occurring sporadically or in the context of von Hippel-Lindau (VHL) disease. They have only been portrayed in isolated case reports and small cohorts. Their natural history and therapeutic strategies are only scarcely described. To better characterize these rare tumors, we retrospectively analyzed an optic nerve and chiasm hemangioblastoma series of 12 VHL patients. By combining our own experience to a review of all known cases in literature, we intended to create treatment recommendations for optic nerve and chiasm hemangioblastomas in VHL patients.

**Methods:**

We reviewed two electronic databases in the hospitals of our senior authors, searching for VHL patients with optic nerve or chiasm hemangioblastomas. Clinical data were summarized. Tumor size and growth rate were measured on contrast enhanced MRI. Comparable data were collected by literature review of all available cases in VHL patients (Pubmed, Trip, Google and Google Scholar).

**Results:**

Of 269 VHL patients, 12 had optic nerve or chiasm hemangioblastomas. In 10 of 12 patients, tumors were diagnosed upon annual ophthalmoscopic/MRI screening. Of 8 patients who were asymptomatic at diagnosis, 7 showed absent or very slow annual progression, without developing significant vision impairment. One patient developed moderate vision impairment. Two symptomatic patients suffered from rapid tumor growth and progressive vision impairment. Both underwent late-stage surgery, resulting in incomplete resection and progressive vision impairment. One patient presented with acute vision field loss. A watchful-waiting approach was adopted because the hemangioblastoma was ineligible for vision-sparing surgery. One patient developed progressive vision impairment after watchful waiting. In the literature we found 45 patient cases with 48 hemangioblastomas.

**Discussion:**

When optic nerve and chiasm hemangioblastomas are diagnosed, we suggest annual MRI follow-up as long as patients do not develop vision impairment. If tumors grow fast, threaten the contralateral eye, or if patients develop progressive vision deficiency; surgical resection must be considered because neurological impairment is irreversible, and resection of large tumors carries a higher risk of further visual decline.

## 1 Introduction

Optic nerve hemangioblastomas are extrinsic primary tumors of the optic nerve, characterized by stromal tumor cells in a rich vascular capillary network (1). They can occur sporadically or as a feature of von Hippel-Lindau (VHL) disease, an autosomal dominantly inherited tumor syndrome. Patients with VHL disease can be affected by central nervous system hemangioblastomas, clear cell renal cell carcinomas, pancreatic neuro-endocrine tumors, pheochromocytomas and other tumors. Retinal hemangioblastomas occur in 49 to 62% of VHL patients and are usually detected at the peripheral retina or at the optic nerve head (2–4). In contrast, the occurrence of hemangioblastomas behind the optic nerve head, thus over the course of the optic nerve and chiasm, is uncommon. These optic nerve hemangioblastomas may be asymptomatic for a long time but can also cause varying degrees of slowly progressive and usually permanent vision impairment (1, 5, 6).

In 1930, Verga et al. were the first to describe a case of a right optic nerve hemangioblastoma in a 57-year-old patient. It is unclear whether this patient had VHL disease. The lesion was described as a “cystic angio-reticulo-glioma” (7).

Up to date, optic nerve and chiasm hemangioblastomas in VHL patients have only been portrayed in isolated case reports and small patient cohorts. It appears that many patients are treated with surgery, leading to further visual decline or blindness. No guidelines exist on the watchful waiting approach.

By combining our own experience to a review of the literature, we intended to create the first algorithm for management of optic nerve and chiasm hemangioblastomas in VHL patients.

## 2 Materials and methods

### 2.1 Patients

**Figure 1:**
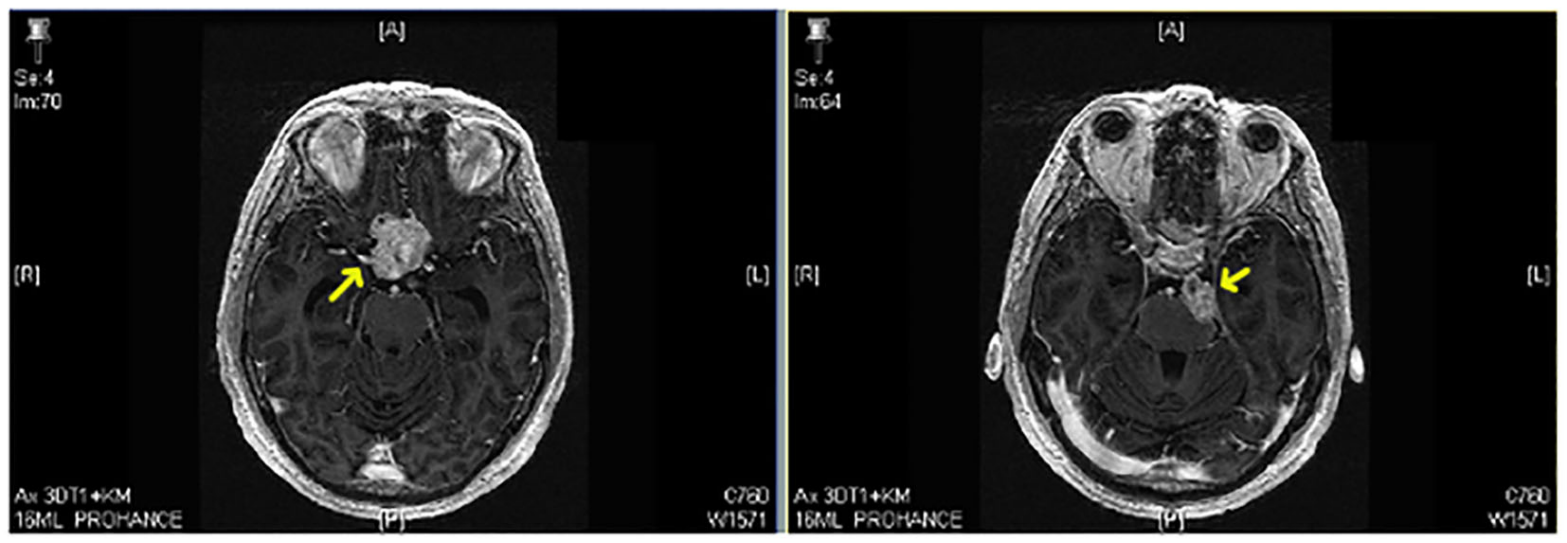
Contrast-enhanced MRI showing an optic nerve hemangioblastoma (yellow arrow) (patient number 12).

We reviewed our databases of 2 hospitals where more than 269 VHL patients with hemangioblastomas of the central nervous system are followed. Patients with optic nerve and/or chiasm hemangioblastomas were included. Clinical data including age, sex, and clinical course (visual ability) were collected. Information about visual ability was derived from ophthalmological and neurological examinations during patient visits. The size of hemangioblastomas, and their location within the optic nerve and chiasm, was assessed on contrast-enhanced magnetic resonance imaging (MRI) (**Figure 1**). The size of the solid tumor (contrast-enhancing region) was measured in three dimensions (height, length, and width), divided by 2 and expressed in cubic millimeter (mm³). Tumor growth rate was measured and expressed as mm³/year.

### 2.2 Review of the literature

Scientific articles on VHL related optic nerve hemangioblastomas were collected by searching through Pubmed, Trip, Google, and Google Scholar databases. We used the following key word combinations: “optic hemangioblastoma”, “optic hemangioblastoma AND von Hippel-Lindau”, “optic nerve AND von Hippel-Lindau”, “optic chiasm AND von Hippel-Lindau”, “ophthalmology AND von Hippel-Lindau”. Also, we reviewed the reference lists of previously published articles on optic nerve hemangioblastomas in VHL patients. Articles on sporadic optic nerve hemangioblastomas were excluded; as decision-making in VHL patients is different and influenced by multi-system comorbidities or the presence of bilateral tumors. We focused on themes such as diagnostic approach, differential diagnosis, surgical pearls and pitfalls, and therapeutic decision making. Histopathologic description of optic nerve hemangioblastomas will not be described here, because more comprehensive papers can be found on this subject.

## 3 Results

### 3.1 Patients from our institutions

Of 269 VHL patients who are followed in the 2 hospitals, 12 patients with optic nerve or chiasm hemangioblastomas were included: 7 male and 5 female patients. One patient was included from a third hospital where one of the authors started to work recently. Age at first diagnosis of an optic hemangioblastoma ranged from 15 to 67 years, with a mean age of 44 years. All included patients had genetically confirmed VHL disease with other intracranial hemangioblastomas. Patient data are summarized in **Table 1**.

**Table 1:** VHL related optic nerve and chiasm hemangioblastomas in our institution: summary of patient and hemangioblastoma data.

No	Age	Sex	Tumor location	Reason of presentation	Treatment	Tumor growth	Later (or postop) visual status
1	42	F	left intraorbital optic nerve	screening: asymptomatic	120m follow-up	0,18 to 0,23mm³ (x1,3/120m or 0,005mm³/y)	reading glasses
2	57	F	left intraorbital optic nerve	screening: asymptomatic	78m follow-up	0,04 to 0,56mm³ (x14/78m or 0,08 mm³/y)	decline
3	30	M	left intraorbital optic nerve, close to chiasm	screening: progressive vision loss left eye	surgery after 12y because of bilateral optic tract edema and threatening of right eye	0,56 to 6,08mm³ (x10,9/144m or 0,46mm³/y)	improvement of right eye
4	50	M	right intracranial optic nerve, close to chiasm	screening: symptomatic	96m follow-up	5,88 to 5,88mm³ (x1/96m or 0mm³/y)	blind
5	20	M	right optic nerve	screening: asymptomatic	52m follow-up	21 to 27,6mm³ (x1,3/52m or 1,52mm³/y)	normal
6	39	M	retrochiasmal	screening: asymptomatic	14m follow-up	0,63 to 0,75 mm³ (x1,3/14m or 0,10mm³/y)	normal
7	45	F	retrochiasmal	screening: asymptomatic	132m follow-up	0,63 to 1,2mm³ (x1,9/132m or 0,052mm³/y)	normal
8	15	F	retrochiasmal	screening: asymptomatic	14m follow-up	2,16 to 5,27mm³ (x2,4/14m or 2,67mm³/y)	normal
9	40	F	retrochiasmal	screening: asymptomatic	48m follow-up	0,38 to 0,90mm³ (x2,4/48m or 0,13mm³/y)	normal
10	66	M	right intracranial optic nerve, close to chiasm	screening: asymptomatic	follow-up	50mm³	normal
11	56	M	right intracranial optic nerve, close to chiasm	acute visual field defect right eye	no effect of corticosteroids, 14m follow-up	1,04 to 1,04mm³ (x1/14m or 0mm³/y)	decline
12	67	M	chiasm	right eye blind except central fovea, left eye superior visual field loss	surgery	52,2mm³	decline

F, female; M, male; m, months; y, years.

### 3.2 Tumor location and growth rate

**Figure 2:**
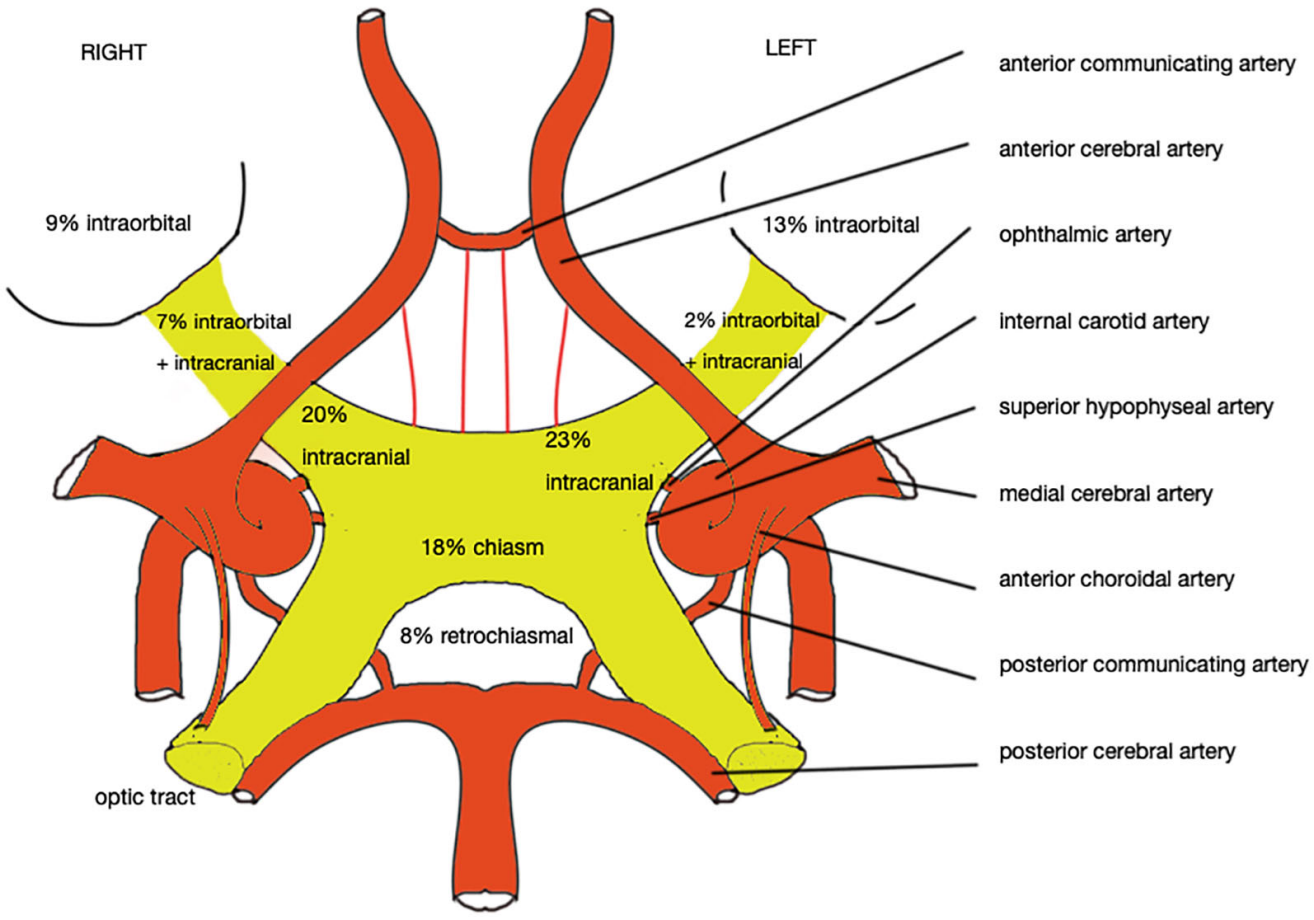
Schematic vascular supply of the optic nerve and chiasm, and the relative distribution of tumors from all available cases (design by E. Vergauwen).

In our patient series, 3 hemangioblastomas were located along the left optic nerve (all 3 intraorbital and 1 with intracranial extension), and 4 hemangioblastomas were located along the right optic nerve (all 4 intracranial). There was 1 hemangioblastoma within the chiasm, and there were 4 with a retro-chiasmal location. Location of tumors is graphically illustrated in **Figure 2**. Mean tumor size at time of diagnosis was 11,23 mm³ (median 0,86mm³). In patients who remained completely asymptomatic during follow up (N=6), tumor size at time of diagnosis ranged from 0,38 to 50mm³ and mean growth rate was 0,89mm³/year (range 0,052 – 2,67mm³/year). In symptomatic patients (N=6), tumor size ranged from 0,04 to 52,2mm³ at presentation and mean growth rate was 0,18mm³/year (range 0 – 0,46mm³/year). Mean growth rate of all tumors (both symptomatic and asymptomatic, N=12) was 0,56mm³/year (range 0 – 2,67mm³/year).

### 3.3 Accessible cases described in literature

Literature cases are summarized in **Table 2**. Literature search yielded 45 patient cases with 48 hemangioblastomas, some cases having been published twice in different years (asterixis in **Table 2**). Most authors presented a single case. One cohort of 19 patients was split up in different publications (Meyerle et. al). Between 1930 and now, 27 female and 28 male VHL patients with optic nerve and/or chiasm hemangioblastomas have been reported, their ages ranging from 15 to 64 years old. Of 48 hemangioblastomas, 17 were located on the right optic nerve (5 intraorbital, 7 intracranial and 5 with extension towards both sides), and 18 were located on the left optic nerve (5 intraorbital, 13 intracranial). There were 3 patients with bilateral optic nerve tumors and 6 patients with tumors of the chiasm. In 1 patient, tumor location was unknown. Location of tumors is graphically illustrated in **Figure 2**. At time of diagnosis, 11 patients were asymptomatic, 27 patients complained of varying ophthalmologic and/or systemic symptoms. In 7 patients, symptomatology is unknown.

**Table 2:** VHL related optic nerve and chiasm hemangioblastomas reported in literature.

Author	Age at presentation in author clinic	Sex	Tumor location	Reason of presentation	VA at time of presentation	Fundoscopy at time of presentation	Visual fields at time of presentation	Evolution	Initial tumor size	Treatment	Later (or postop) tumor size	Months of follow-up	Later (or postop) visual status
*Montoya et al. 2024 *(8)	42	F	R IO optic N, extension into canal and chiasm	cerebellar tumor	NA	other retinal HB	NA	NA	NA	radiosurgery	NA	NA	NA
*McGrath 2018 (* 9)	25	F	R IO optic N	diagnosis on MRI during treatment of retinal HB	RE and LE 6/6	R optic N swelling, bilateral retinal HB coagulation scars	NA	3y: stable vision and MRI. Progressive visual field and VA loss: in 6m, RE and LE 6/18 to only hand movement, right superotemporal field defect, bilateral tract edema on MRI.	3x8mm	no effect of peroral steroids, right lateral orbitotomy	complete tumor resection	6m	no perception of light
*Kanno 2018* (10)	36	F	L IO optic N	recent progressive visual loss LE	RE 0,1; LE sensus luminis	LE pale papilla	narrowing	immediate therapy	NA	stereotactic radiosurgery	slight decrease	18m	blind, no recurrence
*Turkoglu 2016* (11)	30	F	L IO optic N, nerve head	screening: asymptomatic	RE and LE 20/20	LE optic N HB	NA	1y: stable vision. Spontaneous devascularization and detachment.	NA	none	spontaneously detached tumor	immediate	stable vision
*Fard 2014* (12)	39	M	L IC optic N, paraclinoid	2y ago: progressive visual loss LE; steroids for other indication: no effect.	LE light perception	LE optic N head regressed angioma	NA	refused therapy	NA	none	NA	NA	NA
			R IO optic N	1y ago: progressive visual loss RE	RE finger counting	RE optic N head angioma + macular lipid exudates	NA	refused therapy	NA	none	NA	NA	NA
*Staub 2014* (13)	34	F	R IC optic N, distal to chiasm, extension into canal/orbit	5y ago: asymptomatic. Retrospective MRI: tumor size half of size at symptomatic stage, no optic tract edema.	unknown	unknown	NA	progressive right retro-orbital pain, proptosis, RE blind from removal retinal hemangioblastomas, MRI: bilateral optic tract edema	12mm	frontotemporal craniotomy	complete excision of tumor and nerve	NA	no pain
*Zywicke 2012†* (14)	50	F	L IC optic N, extension into canal/paraclinoid	2y: progressive visual loss LE	RE 20/50, LE 20/70	normal	full to confrontation	immediate therapy	10x10mm	left frontotemporal craniotomy	partial excision of tumor, no entrance in optic canal	immediate	LE hand movement and visual field loss
*Prabhu 2009* (15)	32	M	R IC optic N, junction with chiasm	8m: progressive visual loss RE	RE 6/60	primary optic atrophy	RE temporal field defect	immediate therapy	35mm	right suprabrow craniotomy	complete excision of tumor	NA	stable
*Baggenstos 2008* (16)	62***	M	L IC optic N, just proximal to canal	< 24h: rapid visual loss LE	RE 20/16, LE counting fingers	normal	NA	immediate therapy	unknown	IV steroids: subjective improvement, sublabial extended transsphenoidal surgery	complete excision of tumor	immediate	VA LE 20/320
*Barrett 2008* (17)	47	M	R IO optic N, extension into canal	visual loss RE, 5mm proptosis: initial diagnosis of optic N glioma; patient chose watchful waiting	RE light perception	optic nerve pallor	NA	6 y: 11mm proptosis, RE no light perception, growth on MRI	24x40mm	craniotomy + orbitotomy	complete excision of tumor + part of the nerve	NA	NA
*Meyerle 2008* (18)	24 ± 14												
	60	F	R IO optic N	previous enucleation LE; 4y ago: VF defects: diagnosis	RE 20/50	optic nerve pallor	profound loss, sparing superonasal quadrant	2y: stable vision/MRI. 3y: VF defects slightly worsened, died from metastatic pheochromocytoma	18x20x21 mm	follow-up	follow-up	36m	decline
	15	F	L IC optic N, close to chiasm	screening because of multiple retinal HB: asymptomatic	RE and LE 20/30	multiple retinal HB, photocoagulation RE and LE	diminished	6m: growth on MRI, compression of chiasm	9x14mm	frontotemporal craniotomy + transnasal-transsphenoidal surgery	complete excision of tumor	92m	VA RE and LE 20/20, superior nasal quadrant defect
	54***	M	L IC optic N, intracanalicular, edema extension to chiasm	screening	RE and LE 20/20	RE small laser scar, temporal of fovea	unreliable: high fixation loss in RE and LE	stable vision. 8y: acute decline LE to counting fingers. MRI: stable tumor size, increased edema.	7x10mm	IV steroids: no effect on chiasmal edema. transnasal-transsphenoidal surgery	complete excision of tumor	191m	VA RE 20/20, LE 20/320, no progression
	29**	F	L IC optic N, close to chiasm	screening: asymptomatic	RE and LE 20/20	optic disc RE: retinal capillary HB	full to confrontation	immediate therapy	10x8x8mm	frontotemporal craniotomy	nearly complete excision of tumor	172m	VA RE 20/15, LE 20/20, temporal VF defect/optic nerve pallor. MRI stable residual tumor.
*Alvarez et al. 2021* (19)	42 ± 12	11 M, 7 F										115 ± 67m	
	NA	NA	R IC optic N, extension cavernous sinus	trigeminal neuralgia and retro-orbital pain	NA	NA	NA	NA	1468mm³	stereotactic radiosurgery	diminished size with resolution of cyst	81m	persistent neuralgia and pain
	NA	NA	L IC optic N, canal	visual loss	diminished	NA	diminished	NA	583mm³	frontotemporal craniotomy	incomplete excision of tumor	64m	improved VF, persistent VA deficits
	55	M	L IC optic N, canal	few months: LE dimming and blurring	LE 20/20	optic nerve pallor	mean deviation -10,44	2m: LE VA 20/32, VF mean deviation -13,48	248mm³	lateral supraorbital craniotomy	complete excision of tumor	1m	VA 20/32, VF mean deviation -2,99
	NA	NA	L IC optic N, canal	visual loss	diminished	optic atrophy	NA	NA	846mm³	enucleation + frontotemporal craniotomy + stereotactic radiosurgery	incomplete excision of tumor, diminished size after radiation therapy	171m	blind, no further symptoms
	NA	NA	IC, optic chiasm	visual loss	RE diminished	NA	NA	NA	356mm³	none: stereotactic radiosurgery recommended	progression	110m	worsening VA, VF loss
	NA	NA	IC, optic chiasm, suprasellar	visual loss	NA	NA	diminished	NA	1095mm³	monthly IM octreotide	no progression	220m	stable VF deficit, no progression
	NA	NA	IC, optic chiasm, infundibulum	visual loss	diminished	NA	NA	NA	251mm³	follow-up	no progression	128m	worsening VA
	NA	NA	IC, optic chiasm, infundibulum	screening: asymptomatic	NA	NA	NA	NA	138mm³	follow-up	no progression	193m	stable
	NA	NA	IC, optic chiasm	screening: asymptomatic	NA	NA	NA	NA	183mm³	follow-up	no progression	73m	stable
	NA	NA	L IC optic N	screening: asymptomatic	NA	NA	NA	NA	517mm³	follow-up	no progression	140m	stable
	NA	NA	L IC optic N, canal	screening: asymptomatic	NA	NA	NA	NA	102mm³	follow-up	progression	152m	stable
	NA	NA	L IC optic N, canal	screening: asymptomatic	NA	NA	NA	NA	203mm³	follow-up	no progression	38m	stable vision, progression on MRI with edema
	NA	NA	R IC optic N	screening: asymptomatic	NA	NA	NA	NA	805mm³	follow-up	no progression	13m	stable
	NA	NA	R IC optic N, canal	NA	NA	NA	NA	NA	NA	NA	NA	lost to follow-up	
*Higashida 2007†* (20)	64	M	L IO optic N	5y: progressive visual disturbances and VF narrowing, proptosis LE	LE hand motion limited to central vision	NA	narrowing	follow up: “growth”. LE hand motion	NA	left fronto-orbital craniotomy	incomplete excision of tumor	immediate	stable
*Fons Martinez 2006* (21)	35	M	both optic N, R tumor IC extension to chiasm	first: blurred vision RE, VA RE and LE 1, and 2 retinal HB. After 6m: VA RE 0.6, LE 1, cryotherapy RE. After 1y: RE finger counting at 50cm. After 2y: LE visual loss, photocoagulation retinal angioma and diagnosis bilateral optic N HB.	RE finger counting, LE blind	NA	NA	4y: VA RE hand movement, LE 1. 7y: VA RE amaurotic, LE 0.6, bilateral pale papilla, LE central defect extending to nasal hemifield	NA	follow-up	follow-up	84m	“decline”
*Kouri 2000* (9)	20	F	tuber cinereum, posterior to chiasm	secondary amenorrhea, polydipsia	NA	NA	NA	immediate therapy	12x15mm	modified transsphenoidal approach	complete excision of tumor	53m	no visual complications mentioned
	15	F	L IC optic N, close to chiasm	screening: asymptomatic	NA	multiple retinal hemangiomas	NA	immediate therapy	7x14mm	modified transsphenoidal approach	complete excision of tumor	12m	no visual complications mentioned
*Restrepo 2003* (22)	33	F	R IO optic N	3y: proptosis RE	RE and LE 20/25	RE disc elevation	peripheral depression RE, superior arcuate defect LE	6m: VA RE 20/200, swollen and pale disc	NA	craniotomy	complete excision of optic nerve	NA	NA
*Raila 1997* (23)	30**	F	L IC optic N	asymptomatic, development of visual complaints during screening period	RE and LE 20/20 at beginning of screening	RE retinal angioma	normal	immediate therapy	10x8x8mm	surgery	complete excision of tumor	5m	20/15–2RE, 20/20–2 LE
*Kerr 1995* (24)	40	F	R IC optic N	NA	NA	NA	NA	NA	0,50 ml	surgery	NA	24m	decline
*Balcer 1995* (25)	21	F	L IC optic N	2 y: LE progressive difficulty reading	RE 20/15, LE 20/40 + 1	anomalous R optic N, disc atrophy L optic N	bitemporal hemianopsia	immediate therapy	18x13x22 mm	left craniotomy	complete excision of tumor	4m	20/20 RE, 20/40 LE
*Miyagami 1994* (26)	NA	NA	NA	NA	NA	NA	NA	NA	10mm	surgery	NA	60m	NA
*Rubio 1994* (27)	43	F	R IO optic N and canal	4 y: progressive visual loss RE	unknown	unknown	unknown	sudden severe visual loss RE, RE absent pupillary light reflex	20x6mm	surgery, approach unknown	complete excision of tumor and optic nerve	NA	NA
*Ginzburg 1992* (28)	44	M	both optic N, close to chiasm	8m: progressive visual loss RE and LE	“marked reduction” LE>RE	bilateral optic disc pallor	NA	immediate therapy	2 10mm round lesions	surgery	complete excision of R tumor, incomplete excision of L tumor	immediate	RE able to distinguish objects, LE no light perception
*Hotta 1989* (29)	36	M	R IO optic N	3 y: progressive visual loss and proptosis RE	NA	disc atrophy	NA	immediate therapy	16x8x8mm	right frontal extradural craniotomy	NA	NA	NA
*Nerad 1988* (5)	18	F	L IO optic N	diplopia 3w after cerebellar surgery, VA RE 20/30, LE 20/20, bilateral papilledema, resolved in few months. 7y later: progressive visual loss LE, proptosis.	RE 20/20, LE 20/60	LE swollen disc	inferior visual field defect	11m: incisional biopsy via lateral orbitotomy without visual decline (LE 20/200). 2y: blind, exotropia, proptosis.	almost complete retrobulbar filling of orbit on CT	lateral orbitotomy	complete excision of tumor and optic nerve	5m	blind, no proptosis
*Tanaka et al. 1984* (30)	37	M	R IO optic N	NA	NA	NA	NA	NA	10cm³	NA	NA	NA	NA
*In 1982*	23	F	L IO optic N and canal	7y: visual blurring to progressive blindness, pulsating exophthalmos	LE absent direct light reflex	angiomatosis retinae	blind	immediate therapy	29x17x15 mm	left frontal osteoplastic craniotomy	complete excision of tumor and optic nerve	36m	blind
*Lauten 1981†* (31)	15	M	L optic N, canal, intracranial bulging	6m: visual loss and proptosis LE	LE light perception only	LE pale disc	NA	immediate therapy	15x5x3 mm	craniotomy	complete excision of tumor and optic nerve	immediate	blind, no proptosis
*Uehara and Ichinomiya 1974 *(32)	20	M	R IC optic N	NA	NA	NA	NA	NA	7,4cm³	NA	NA	NA	NA
*Stefani 1974† (* 33)	43	M	R IC optic N, close to chiasm	VA RE never as good as LE. 6m: RE accelerated visual loss, headache, feeling of pressure.	RE 5/35, LE 5/7.5	RE swollen elevated optic disk	RE concentric narrowing	few months without progression, “later”: blindness. Died of cardiac failure 23y later. Ophthalmoscopy: total atrophy R optic N.	11x9mm	follow-up	NA	NA	NA
*Verga 1930 (* 34)	57	F	R IC optic N, close to chiasm	NA	autopsy	NA	NA	NA	NA	NA	NA	NA	NA

** same patient, *** probably same patient, † probably no VHL, F, female; M, male; L, left; R, right; IO, intra-orbital; IC, intracranial; N, nerve; HB, hemangioblastoma; LE, left eye; RE, right eye; NA, not available; ref., refraction; VA, visual acuity; VF, visual field; w, weeks; m, months; y, year.

### 3.4 Clinical outcomes of own cases and cases in literature

**Figure 3:**
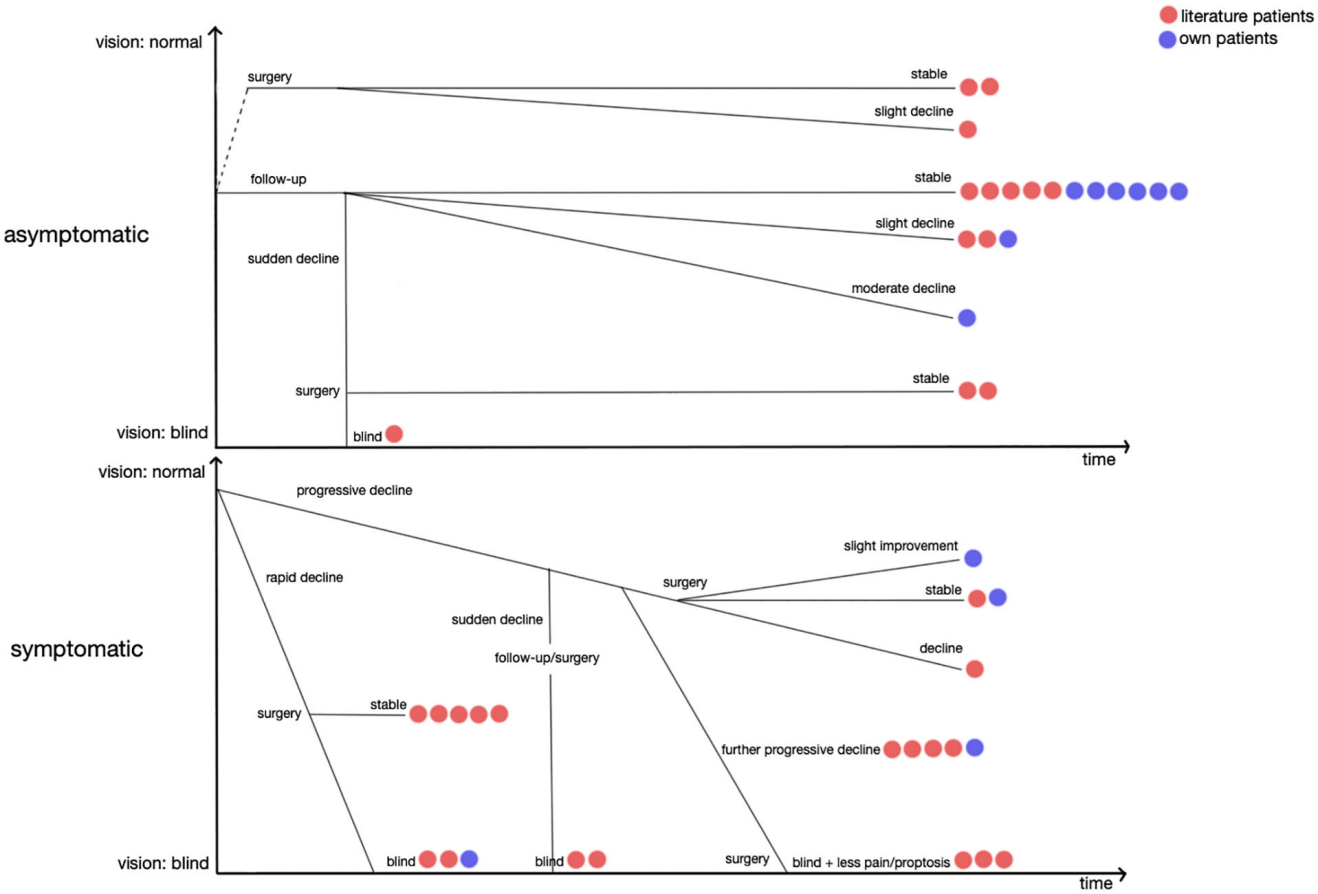
Possible clinical scenarios in patients with optic nerve hemangioblastomas. (Red dots = own patients. Blue dots = literature patients with available progression data. One dot represents one patient. Horizontal axis = progression over time. Vertical axis = vision, blind to normal).

In the literature we found 45 cases of VHL patients with 48 optic nerve and chiasm hemangioblastomas. Often the key message of these case reports was that the discovery of a rare optic nerve tumor led to diagnosis of VHL disease. Of 45 reported patients, 24 underwent surgical resection, 4 were treated with stereotactic radiosurgery, 13 received no treatment other than pharmacological, and in 4 patients, treatment modality is unknown. **Figure 3** is a graphical illustration of the different clinical scenarios in our own patients and the scenarios that could be reconstructed from literature cases. These possible scenarios are further described in the following section.

#### 3.4.1 Asymptomatic patients who remained asymptomatic with watchful waiting

Of 8 asymptomatic patients followed in our clinic, 6 patients remained asymptomatic during a mean watchful waiting period of 4,3 years (range 1,17–11 years). Mean tumor growth rate was 0,89 mm³/year (range 0,052 – 2,67mm³year).

In literature, 6 asymptomatic patients remained asymptomatic during varying follow-up periods. Remarkably, Turkoglu et al. observed spontaneous devascularization and detachment of an optic nerve head hemangioblastoma in a 30-year-old asymptomatic female patient, after 1 year of watchful waiting (11).

#### 3.4.2 Asymptomatic patients who became symptomatic with watchful waiting

On annual MRI screening, we discovered an intraorbital left optic nerve hemangioblastoma in a 42-year-old asymptomatic female patient. During a follow-up time of 8 years without intervention, the tumor grew from 0,18 to 0,23mm³ (0,006mm³/year) on MRI, and the patient only needed reading glasses.

Also on annual MRI screening, we discovered an intraorbital left optic nerve hemangioblastoma in a 57-year-old asymptomatic female patient. After 6,5 years of watchful waiting, the tumor grew from 0,04 to 0,56mm³ (0,08mm³/year) and visual acuity in the left eye was +4,75/-0,25.

#### 3.4.3 Asymptomatic patients who underwent surgery

Meyerle et al. performed complete resection of an intracranial left optic nerve hemangioblastoma in a 29-year-old asymptomatic female patient. Postoperatively, the patient experienced slight visual decline in both eyes (18).

Kouri et al. performed complete transsphenoidal resection of a suprachiasmal hypothalamic hemangioblastoma in a 20-year-old female patient with hormonal changes and normal vision. Her vision remained stable after surgery. Kouri et al. also reported the case of a 15-year-old asymptomatic female patient with preservation of normal vision after complete transsphenoidal resection of a left optic nerve tumor close to the chiasm (9).

#### 3.4.4 Symptomatic patients who slowly progressed to (near) blindness with watchful waiting

In our clinic, we followed a 50-year-old male patient with a right optic nerve hemangioblastoma close to the chiasm. The patient experienced visual complaints in the beginning and became progressively blind after a watchful waiting period of 8 years, although no growth was observed on MRI.

Meyerle et al. reported a 60-year-old female patient with a right intraorbital optic nerve hemangioblastoma. The patient experienced progressive visual field defects in the right eye, and after a watchful waiting period of 4 years, the optic nerve had a pale fundoscopic aspect. Her vision remained stable in the sixth and seventh year, but again mildly declined during the eighth year (18).

Fons Martinez et al. discovered bilateral optic nerve hemangioblastomas in a 35-year-old male patient who already had bilateral retinal hemangioblastomas. The patient could only count fingers with the right eye and had normal vision in the left eye. After 4 years of watchful waiting, his already poor left eye vision remained stable, but his right eye vision regressed to perceiving hand movements. After another 3 years, vision further declined in both eyes, and fundoscopy showed bilateral optic disc pallor (21).

Fard et al. reported a 39-year-old male patient who experienced bilateral progressive vision impairment over 2 years; to the point of finger counting in the right eye, where he had an intraorbital optic nerve hemangioblastoma; and of light perception in the left eye, where he had an intracranial optic nerve hemangioblastoma. The patient refused therapy (12).

Staub et al. presented a similar case of a 34-year-old female patient, who had both retinal hemangioblastomas and an intracranial nerve hemangioblastoma in the right eye. The hemangioblastoma doubled in size over a 5-year period, during which her right eye vision evolved from normal to blind. Because of acute and rapidly progressive pain with bilateral white tract edema on MRI, the hemangioblastoma together with the right optic nerve were completely resected. Vision in the left eye was spared and pain resolved (13).

#### 3.4.5 Symptomatic patients who slowly progressed to (near) blindness, and with further visual decline after surgery

Zywicke et al. followed a 50-year-old female patient who had an intracranial left optic nerve hemangioblastoma that extended into the optic canal. The patient experienced bilateral progressive vision impairment over a 2-year period. After the intracranial part of the tumor was resected, she experienced visual decline in the left eye, but right eye vision remained stable (14).

#### 3.4.6 Symptomatic patients with slow progression, and stable/improved vision after surgery

We followed a 30-year-old male patient with progressive vision impairment in the left eye because of a left optic nerve hemangioblastoma close to the chiasm. After 12 years of watchful waiting, the tumor had grown from 0,56 to 6,08mm³ (0,46mm³/year) on MRI, and the left optic disc had become pale on fundoscopy. During the twelfth year, the patient developed a visual field defect in his other – right – eye. MRI showed tumor and edema progression from the left optic hemangioblastoma to the right optic nerve, as far as the geniculate bodies. In order to prevent further spread of edema, the patient underwent complete surgical resection of the hemangioblastoma. Bilateral visual acuity slightly decreased after surgery. Visual field restriction improved in the right eye, and slightly deteriorated in the left eye.

We followed a 67-year-old male patient with a large chiasmal hemangioblastoma that extended to both optic nerves. The patient was almost blind in his right eye but nevertheless he was operated because of progressive visual field loss in the left eye. Visual status remained stable after complete left-sided and incomplete right-sided resection of the tumor.

In 1995, Balcer et al. reported a 21-year-old female patient with an intracranial left optic nerve hemangioblastoma, who experienced bilateral progressive slight visual decline over 2 years. After total excision of the left optic nerve tumor, she experienced bilateral mild visual improvement (25).

#### 3.4.7 Asymptomatic/symptomatic patients with slow progression and then sudden decline

Rubio et al. reported a 43-year-old female patient with an intraorbital right optic nerve hemangioblastoma that extended into the optic canal. After 4 years of slight visual decline in the right eye, she experienced sudden blindness of the right eye. The right optic nerve was completely resected, resulting in blindness of the right eye (27).

McGrath et al. published a case of a 25-year-old asymptomatic female patient with a right intraorbital optic nerve tumor. For 3 years she remained clinically and radiographically stable, until rapid visual decline developed over a six-month period, due to bilateral optic tract edema that could be seen on MRI. The tumor was completely resected and postoperatively the patient was only able to discern light in the right eye (35).

Meyerle et al. reported a 54-year-old male asymptomatic patient with a left intracranial optic nerve hemangioblastoma extending to the optic canal, who remained stable for 8 years, until his vision suddenly declined to counting fingers only. As in McGrath’s case, bilateral white tract edema was seen on MRI. The tumor was completely resected, resulting in slight visual improvement in the left eye and sparing of vision in the right eye (18).

#### 3.4.8 Symptomatic patients with acute and rapid progression

Kanno et al. wrote a case report on a 36-year-old female patient, who had rapid visual decline until the left eye could only perceive light and the optic disc became pale on fundoscopy. A large left intraorbital optic nerve hemangioblastoma was discovered. The patient became completely blind after stereotactic radiosurgery (10).

In 1992, Ginzburg et al. reported a 44-year-old male patient who experienced severe bilateral vision impairment over an 8-month period, due to bilateral optic nerve hemangioblastomas. Partial tumor resection in the left eye led to blindness, and total tumor resection in the right eye led to being able to distinguish objects (28).

Prabhu et al. described a 32-year-old male patient, with a right intracranial optic nerve hemangioblastoma that caused visual decline in the right eye over an 8-month period. Vision remained stable after complete tumor resection (15).

Baggenstos et al. reported a 62-year-old male patient with an intracranial left optic nerve hemangioblastoma with bilateral white tract edema, who experienced progressive vision impairment in the right eye and sudden vision impairment in the left eye. After complete resection of the tumor, edema resolved and left eye vision improved (16).

Meyerle et al. presented a 15-year-old female patient who had no visual complaints; however, there was slight vision loss in the left eye on measuring. She had an intracranial left optic nerve hemangioblastoma that radiologically grew over a 6-month period and then started to compress the chiasm. The tumor was completely resected, and after 9 years, the patient had a small quadranopsia but regained visual acuity (18).

**Figure 4:**
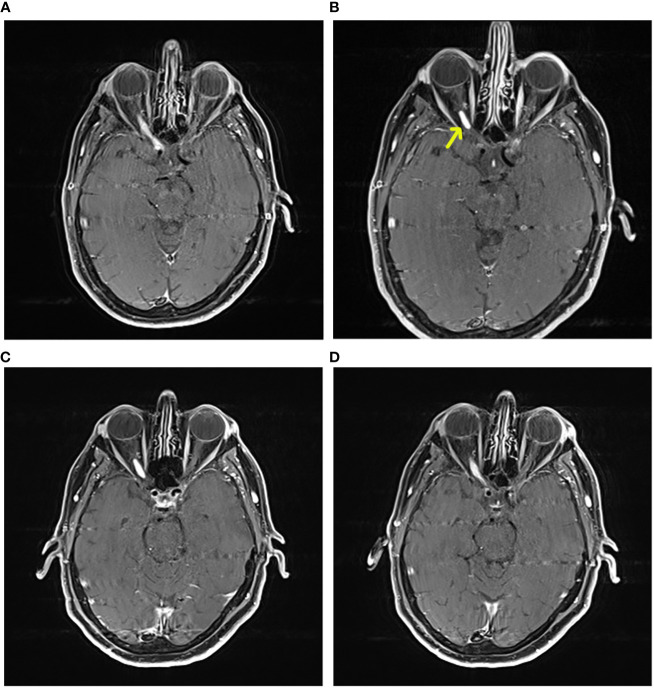
Axial contrast enhanced T1 MRI. A 56-year-old male VHL patient followed in our clinic with a right optic nerve hemangioblastoma. The patient experienced acute visual field defects in the right eye. Fundoscopy was normal. MRI showed a large right intraorbital optic nerve hemangioblastoma without edema (yellow arrow). Corticosteroids were not effective for reducing symptoms. Because surgical resection was unfeasible and was anticipated to lead to total blindness of the right eye, the patient was not operated – to cherish a couple more “good” years. Surgery will be planned as soon as peritumoral edema develops and threatens the contralateral eye. Tumor volume has now remained stable for 4 years. **(A)** time at diagnosis, tumor length 13mm and width 4mm. **(B)** 20 months later, tumor length 31mm and width 6.7mm (= growth); note progression towards optic chiasm, and less intense contrast enhancement, compared to **(A, C)**: also 20 months, but shorter image acquisition time after contrast administration, now there is no growth compared to **(A, D)**: longer image acquisition time after contrast administration, tumor length 24mm, width 6mm, there is growth compared to **(A)** Note peritumoral contrast leakage along optic nerve tract, similar to **(B)**.

A 56-year-old patient in our clinic experienced an acute visual field defect in the right eye. Fundoscopy was normal and MRI showed a large right intraorbital optic nerve hemangioblastoma without surrounding edema (**Figure 4**, Discussion). Corticosteroids did not improve his symptoms. Because surgical resection was unfeasible and was anticipated to lead to total blindness of the right eye, the patient was not operated – to cherish a couple more “good” years. Surgery will be planned as soon as peritumoral edema develops and threatens the contralateral eye. Tumor volume has now remained stable for 4 years.

According to both literature and our own experience, sudden visual decline is often related to rapid development of (bilateral) white matter tract edema. Also, progressive disk swelling appears to be a red flag. Restrepo et al. reported a 33-year-old female patient with an intraorbital right optic nerve hemangioblastoma, who experienced bilateral progressive slight visual decline and proptosis over a 3-year period. What started as a right optic disc elevation, further evolved to a swollen and pale disc until the patient experienced sudden deterioration of vision in the right eye. The right optic nerve was completely resected and the patient became blind in her right eye (22). Stefani et al. published a similar case report on a 43-year-old male patient with an intracranial right optic nerve hemangioblastoma. The moment when the swollen optic disk became atrophic on fundoscopy, the patient experienced rapidly progressive blindness of the right eye over less than one year (33).

#### 3.4.9 Clinical course unknown

In some case reports, there is no focus on symptomatology or it is not mentioned (Kerr et al. (24), Hotta et al. (29), Miyagami et al. (26), Tanaka et al. (30), Uehara and Ichinomiya (32) and Verga et al. (7), Vásquez Montoya et al. (8).

### 3.5 Review of the literature

#### 3.5.1 Symptomatology

Optic nerve hemangioblastomas cause symptoms by compression, displacement, and ultimately replacement of native optic nerve fibers and their vasculature (22, 36). We found 7 case reports on patients who were asymptomatic at time of diagnosis; hence, hemangioblastomas were discovered on annual MRI screening. Some hemangioblastomas were discovered by screening the family members of VHL patients (9, 11, 18, 23). All other reported patients were symptomatic at time of diagnosis. The time interval between initiation of symptoms and the seeking of medical attention ranged from 24 hours to 7 years.

Patients complain of sudden or gradual “vision loss” (5, 10, 12, 14–18, 20, 21, 25, 27–29, 31, 33), blurry vision (37), difficulty reading (25) or narrowing of the visual field (10, 20). Sometimes patients become progressively or very suddenly blind (28, 37). Other patients experience headache or a feeling of pressure (33). Rarely, hypopituitarism due to pituitary compression leads to diabetes insipidus with polydipsia, irregular menses, or secondary amenorrhea (9, 25).

#### 3.5.2 Physical and technical examination

A fairly large group of patients suffers from painful or painless (pulsating) proptosis with secondary lagophthalmos and corneal epithelial exposure changes (5, 13, 17, 18, 20, 22, 29, 31, 37). Ocular movement may be normal (14, 20), or patients may have motility disturbances such as skew deviation or exotropia (5). Intracanalicular compression may cause hypoesthesia in the V1 dermatome (5). Patients with advanced optic nerve or anterior optic pathway destruction may have a sluggish pupillary reaction to light (12), an afferent pupillary light defect (16, 22, 25), a relative afferent pupillary light defect (5, 12, 31, 35), or a dilated pupil without direct light reflex (20, 21, 27, 37). Visual acuity may range from normal to blind. Color vision may be diminished; this is not the case with most hemangioblastomas of the retina in VHL disease (16, 18). Visual fields may be full to confrontation (14, 18, 23). On formal visual field testing, various defects may be encountered: monocular blindness, concentric field narrowing (20, 33), quadranopsia (35), central or arcuate monocular defects (5, 15, 18, 21, 22, 35), peripheral field narrowing (22) or bitemporal hemianopia (18, 25). In our clinic we sometimes use optical coherence tomography (OCT) to visualize thinning of the ganglion cell layer secondary to central lesions causing visual field defects. Visual evoked potentials may be decreased or absent (27).

#### 3.5.3 Ophthalmoscopy

Fundus examination is normal in some patients (14, 16, 18, 23). When they are located in the anterior optic nerve, or just posterior to the lamina cribrosa, optic nerve hemangioblastomas cause optic disc elevation (5, 22, 33), progressive disc pallor (10, 17, 18, 21, 22, 28, 31), disc atrophy (5, 15, 25, 29, 33), pseudo-papilledema (false swelling), or anterior optic neuropathy due to infiltration. When located more posteriorly in the optic nerve, hemangioblastomas cause true swelling of the optic nerve and a posterior optic neuropathy (1, 6). Retinal pigment epithelium granularity may have an abnormal aspect, and epiretinal membranes or choroidal folds may be seen (18). Bilateral papilledema is caused by either bilateral optic hemangioblastomas or by edema spread through the chiasm.

Fluorescein angiography may be useful for discovery of optic nerve head hemangioblastomas, by showing early hyper-fluorescence and venous staining (12), or by directly exposing the tumors (21). VHL patients may simultaneously suffer from retinal and optic nerve hemangioblastomas; hence, making it difficult to identify the cause of vision loss. Clinicians need to be aware that retinal hemangioblastomas may mask and delay the discovery of intraorbital and intracranial optic nerve hemangioblastomas. The presence of an optic nerve hemangioblastoma should be considered when the type of vision disturbances cannot be explained by retinal lesions (18), and in the absence of visual improvement after photocoagulation. Also, previous enucleation of the eye due to large retinal lesions does not exclude the development of a hemangioblastoma along the course of the optic nerve, which can potentially harm the other eye by edematous spread.

#### 3.5.4 Laboratory examination

In et al. mentioned normal blood biochemical analysis in their patient (37). Rubio et al. reported an erythrocyte sedimentation rate of 40mm/h in a patient with a right optic nerve hemangioblastoma. However, the patient had VHL disease with multi-organ involvement that may have interfered with the result. According to our own previous research, hematocrit may be high due to tumoral erythropoietin production that may be seen in hemangioblastomas (38).

As described in two cases, we recommend testing of pituitary function before surgery if the optic nerve hemangioblastomas is in close contact to the pituitary stalk or gland (9, 25).

Stefani and colleagues reported normal spinal fluid analysis in one patient. We found no other data on spinal fluid analysis in the literature (33).

#### 3.5.5 Radiographical features

On computed tomography (CT) imaging, optic nerve and chiasm hemangioblastomas appear isodense compared to normal brain parenchyma and show moderate or dense and diffuse enhancement after iodinated contrast administration (1, 22, 28, 29, 35). They can result in enlargement and/or kinking of the optic nerve or chiasm (17, 22). Tumors can be found at the optic nerve head, along the course of the optic nerve or at the chiasm, and therefore they can be situated intraorbitally, intracranially or both. They can be located on the sheath or the center of the optic nerve (22). On coronal CT images, optic nerve hemangioblastomas cannot be clearly distinguished from the optic nerve, and just seem to enlarge the optic nerve mass in toto (27, 35). Sometimes, optic hemangioblastomas cause concentric enlargement of the bony optic canal (31). Intraorbital tumors may extend intracranially towards the chiasm (23), or cause erosion of the planum sphenoidale, the anterior clinoid process (12, 14, 23, 31) or the tuberculum sellae (23). On CT, this bony infiltration may be seen as a widened area between the latter two (23). Orbital X-rays or CT may be used for evaluation of bony expansion and surgical planning (5, 22, 29, 37).

Optic nerve hemangioblastomas are usually well-defined solid iso-intense masses on T1-weighted MRI (15, 20, 23), and show intermediate or high signal intensity with varying degrees of homogeneity and vascular flow-voids on T2-weighted MRI (15, 18, 20, 22, 23, 35). If large enough, they intensely and homogeneously stain on contrast enhanced MRI, with clearly depicted borders (9, 10, 12–18, 20–23, 25, 27, 28, 35) and without dural enhancement, in comparison to meningiomas (15). According to our own experience, it may be tricky to distinguish hemangioblastomas from intraorbital fat on contrast enhanced T1-weighted MRI or Fluid Attenuated Inversion Recovery (FLAIR). When in doubt, Short Tau Inversion Recovery (STIR) or fat suppression sequences may be used.

Large peritumoral cysts are less often encountered than with cerebellar hemangioblastomas, which can also be found in VHL patients (20, 22).

Optic nerve hemangioblastomas have immature dilated vasculature, sometimes visible on MRI, with weak tight junctions and secondary leakage of plasma ultrafiltrate. The tumors are usually supplied by the high-flow ophthalmic artery. We believe that secretion of vasoactive substances and leakage of plasma ultrafiltrate may be the cause of the massive interstitial edema that is sometimes seen around the tumor. Edema typically spreads through low-resistance white matter tracts; hence, it preferentially spreads to the chiasm, the optic white matter tracts, the lateral geniculate bodies, the neuronal synapses and the optic radiations (13, 16, 18, 22, 28, 35). This vasogenic edema can have a disproportionately large volume compared to the tumor itself (13). Peritumoral edema has intermediate or high signal intensity on T2-MRI (22). It can be easily seen on FLAIR-images (13, 15, 16), but does not enhance after gadolinium contrast administration (22). Sometimes, edema spreads to the contralateral visual pathways, resulting in bilateral vision loss (13, 16, 28).

On conventional angiography, or CT/MRI angiography, optic nerve hemangioblastomas appear as highly vascular masses (22, 29, 37), showing homogenous staining (31). Tumors can be supplied by the usual vasculature of the optic nerve and chiasm i.e. the ophthalmic artery (17, 20, 22, 23, 29, 37), anterior cerebral artery, anterior communicating artery, superior hypophyseal artery, posterior communicating artery and anterior choroidal artery; or as described by some authors: the meningohypophyseal trunc (23), long posterior ciliary artery (22), superficial temporal artery (17) and internal maxillary artery (17). (**Figure 2**) Optic nerve hemangioblastomas have deep dilated draining veins (20, 31).

In cases where high perioperative bleeding is expected, preoperative embolization of major supplying vessels may be considered, but this is usually not feasible because of shared blood supply with the optic nerve.

#### 3.5.6 Differential diagnosis

In VHL patients, symptomatic retinal and other nervous system hemangioblastomas may lead to delay in diagnosis of an optic nerve hemangioblastoma (21). Also, hemangioblastomas may be mistaken on imaging for retrobulbar optic neuritis (20, 27, 33), optic nerve (microcystic) glioma either related or unrelated to neurofibromatosis type 1 (10, 12, 13, 17, 20–23, 25, 28, 31, 35), optic nerve sheath (angioblastic) clinoid or sphenoid wing meningioma either related or unrelated to neurofibromatosis type 2 (5, 10, 12, 14, 15, 20–23, 25, 27, 31, 37), schwannoma (10, 12, 20), germinoma (25), teratoma (25), craniopharyngioma (25), lymphoma (22, 28), granuloma (12, 22, 28), aneurysm (28), hemangioma (37), and hemangiopericytoma (5). Last, brain metastasis of a clear cell renal cell carcinoma should be considered, especially in VHL patients who are prone for the development of these malignant tumors (12, 17, 22).

The typical growth patterns of the abovementioned other tumors can be used for differentiation with optic nerve hemangioblastomas. Low-grade meningiomas grow slowly while gliomas and especially brain metastasis show rapid progression. It is however frustrating that hemangioblastomas are known for their capricious growth, which may be linear, exponential, saltatory or very rapid after years of stability (39).

Meningiomas can be calcified on CT (22). Optic nerve hemangioblastomas may cause expansion of the bony optic canal on coronal CT. This expansion is benign and usually non-erosive, while brain metastases may cause wide bony destruction (17).

Hemangioblastomas, meningiomas and gliomas can seem hyperintense on T1 MRI (23). Usually, optic nerve gliomas and optic neuritis do not fiercely enhance after contrast administration; whereas meningiomas, brain metastases and hemangioblastomas can enhance brightly; however, hemangioblastomas usually cause the largest mass of edema spreading throughout white matter tracts (17, 20–22, 35). Caution must be taken not to misidentify an infiltrating optic glioma or an astrocytoma from hemangioblastomatous edema or peritumoral gliosis respectively (13, 35).

Vascular flow voids can be seen in hemangioblastomas with thick vessels, but flow voids are absent in gliomas and can only rarely be seen in large meningiomas (18).

In meningiomas, a contrast enhancing dural tail may be seen on coronal MRI images, while hemangioblastomas and brain metastases are located within the nerve (12, 17).

On angiography, both hemangioblastomas and brain metastases seem like highly vascularized masses compared to gliomas (17). However, nevertheless its vascular nature, spontaneous bleeding in a hemangioblastoma is very rare. Nerad et al. suggested incisional biopsy for diagnosis of optic hemangioblastomas in patients with good vision and doubt of diagnosis; however, we do not recommend it because of the high risk of iatrogenic hemorrhage and morbidity. Also, an optic nerve tumor in a known VHL patient, is very likely to be a hemangioblastoma and not another rare optic nerve tumor (5). Conversely, if the patient is not known with VHL disease, the diagnosis of an optic nerve hemangioblastoma warrants screening for VHL disease.

## 4 Discussion

No guidelines exist on the management on optic nerve hemangioblastomas. The authors of the VHL handbook recognize that optic nerve-sparing surgery can be very challenging; thus, they suggest not to operate on asymptomatic optic nerve hemangioblastomas, without further specification on the broad range of possible clinical scenarios (40). By combining our own experience to an in-depth review of all available cases in literature, we intended to create the first algorithm for management of optic nerve and chiasm hemangioblastomas in VHL patients.

### 4.1 Surgical indications

Growth of an optic nerve hemangioblastoma can cause progressive vision impairment and proptosis. Even though optic nerve hemangioblastomas cause only compression and splicing of the optic nerve fibers, complete excision or restoration of lost visual function is usually not possible. Especially if tumors have resided for a long time in the optic nerve, some compressed nerve fibers may have become permanently damaged and replaced by tumor tissue (5, 16, 22, 28).

Postoperative visual prognosis depends on timing of diagnosis, preoperative visual function, tumor size, tumor location, and number of tumors. Postoperative improvement of vision may take a couple of hours to one week if vision loss is caused by peritumoral edema (16, 23, 35). Different authors have reported that the resolution of peritumoral edema may take three days to one week after transsphenoidal removal of optic nerve hemangioblastomas (13, 16, 18). Vision may transiently deteriorate immediately after surgery (23).

The surgical indications we found in the literature included: (1) young age, (2) radiographical growth, (3) postoperative vision loss expected to be minimal, (4) to prevent symptoms in an asymptomatic patient, and (5) to preserve contralateral vision (18). Some authors have stated that watchful waiting always results in vision impairment (21); however, based on our own success with the watchful waiting approach, we cannot agree with this statement. We only recommend considering surgery if patients develop vision impairment, if tumors grow fast, or if there is contralateral spread of either tumor or edema.

Rapid visual decline and bilateral symptoms due to peritumoral edema are major indications for urgent surgery (18, 35, 41). Edema is an early indicator of impending vision disturbance (16).

Most authors recommend complete excision of the optic nerve if vision is already lost and the patient has severe or painful proptosis (5, 35). Incomplete resection of such hemangioblastomas usually leads to recurrence and accelerated growth (22, 42–44). Postoperatively, regression of proptosis may take some months (5).

In VHL patients, contralateral eye function and prognosis of other VHL-associated tumors are also major influencers on surgical decision making.

**Figure 5:**
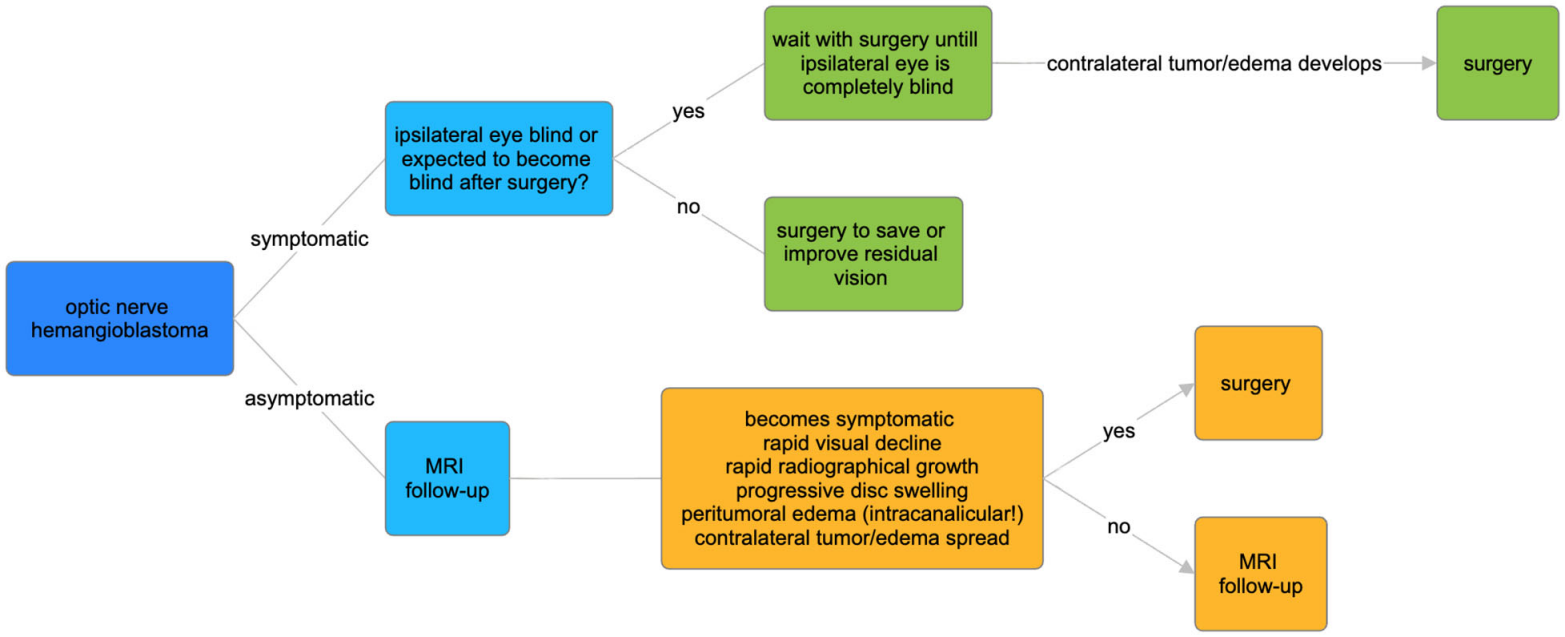
Suggested protocol for treatment and follow-up of VHL optic nerve hemangioblastomas.

A suggested follow-up and treatment protocol is graphically summarized in **Figure 5**.

### 4.2 Surgical technique

Various surgical techniques for removal of optic nerve hemangioblastomas can be found in the literature. The lateral orbitotomy (5, 35), frontal orbitotomy (20) and frontal extradural approach (29) have been described for intraorbital tumors. For intracranial tumors nearby the chiasm, authors have described the suprabrow craniotomy (15), transnasal-transsphenoidal approach (18) and sublabial transseptal transsphenoidal approach (i.e. removing the posterior portion of the planum sphenoidale). The anterior chiasm can also be reached with orbitotomy, while frontal (extradural) craniotomy can be used for posterior intraorbital tumors (37). The fronto-temporal craniotomy (13, 14), mini-pterional craniotomy and transnasal-transsphenoidal approach can be used for intracranial tumors with intracanalicular extension. In order to reach intracranial tumors proximal to the canal, the sublabial extended transsphenoidal approach (16) has been described. Nerad et al. used a CO2 laser to reach intracranially extending orbital tumors (5). By using a frontal approach, the entry of the optic canal can also be exposed (37). However, some neurosurgeons prefer not to disturb canalicular anatomy because of the risk of disrupting the annulus of Zinn where the four rectus muscles originate.

The transsphenoidal access may be valuable for growing children with normal pituitary function because it avoids frontotemporal lobe retraction and separation of the sylvian fissure. Depending on the experience of the surgeon, transsphenoidal surgery is less likely to cause morbidity and mortality. Nevertheless, an large sella is required for broad exposure because internal carotid artery injury is an eminent threat (9).

In the literature, all authors describe optic hemangioblastomas as soft, highly vascular orange-red tumors, with tortuous blood vessels on the surface. Hemangioblastomas are solid or partly cystic. They are not attached to the dura mater and do not have a capsule. The tumor may be centrally or peripherally located in the optic nerve (22). It may be difficult to differentiate tumor from native nerve fibers (20). Sometimes, the tumor is firmly attached to the pial nerve sheet (18). If tumors are completely embedded in the optic nerve, surgeons seem to prefer a longitudinal dissection through the damaged part of the nerve, close to the surface of the tumor, thus minimizing the risk of nerve fiber loss (13, 15, 24). Raila et al. described removal of an optic hemangioblastoma using piecemeal dissection (23); however, we do not recommend it because hemangioblastomas typically bleed profoundly; and to avoid optic nerve ischemia, care should be taken with coagulation. Following other authors, we rather recommend coagulation of the major supplying vessel prior to circumferential dissection from the pia in toto (15, 16). When there is an associated cyst, we suggest to first fenestrate the cyst in order to widen the surgical field and facilitate dissection of the nodule.

We found many literature cases where the optic nerve was resected together with the tumor, resulting in blindness of the eye. Different portions of the optic nerve can be resected: the intraorbital portion up to the entry point in the optic canal, the intracanalicular portion, and the extraorbital portion up to the chiasm. Staub et al. have advised not to transect the optic nerve too close to the chiasm. This action may harm “Wilbrand’s knee”: a loop of decussating fibers that originates from the contralateral optic nerve, and heads to the ipsilateral optic tract. Lee and colleagues believe that this loop is a histological artifact, and that it is only seen in enucleated eyes with secondary optic atrophy of the contralateral eye. We conclude that it seems reasonable to section the optic nerve 1–2mm in front of the chiasm (13, 45); however, there are no studies on optic tractography to support our suggestion (13).

### 4.3 Surgical complications

Optic nerve hemangioblastomas may bleed profoundly, leading to postoperative visual decline. Therefore, we do not recommend performing a biopsy or incision unless the tumor is totally resected (28). Some optic hemangioblastomas may be difficult to access, because of their central location in the optic nerve, or involvement of the surrounding structures such as the internal carotid artery.

It may be challenging to reach tumors inside the optic canal. Even though intracanalicular hemangioblastomas may remain stable for a long time (46), sudden white matter edema and swelling of the optic nerve in this narrow alley may result in rapidly evolving blindness. The intracanalicular and intraorbital portions of the optic nerve are poorly vascularized and collateralized. This natural vascular handicap can be magnified by a steal phenomenon from the hemangioblastoma (47). The intracanalicular portion of the optic nerve is supplied by two or three small trunks from the superior hypophyseal artery, and to a lesser part by the ophthalmic artery; branching into a microscopic dense capillary network that is prone to perioperative bleeding (34, 48). The vulnerability of this vasculature, together with the imminent threat of edema, makes this narrow alley a worthy adversary during surgery.

Other reported surgical complications are eye motility disturbance (5) and transient diabetes insipidus due to hypophyseal traction (23). Transsphenoidal approaches may be complicated by bleeding, cerebrospinal fluid rhinorrhea and meningitis (9).

### 4.4 Follow-up

Contrast-enhanced MRI can be used for follow-up of tumor volume and peritumoral edema. According to our own experience, the typical leaky vasculature of hemangioblastomas may cause contrast extravasation and therefore an overestimation of tumor volume on MRI. We suggest standardizing the time interval between intravenous gadolinium administration and MRI image acquisition, to minimize the effect of this overestimation on tumor volume comparison (**Figure 4**).

### 4.5 Other treatments

We found some case-reports mentioning that high-dose radiation therapy is ineffective for reduction of hemangioblastoma volume (5, 10). We have the same experience, and believe that the use of radiotherapy is not justifiable due to devastating side-effects and potential worsening of ipsilateral or contralateral vision. Stereotactic radiosurgery may be considered in very selected cases with multifocal, nonresectable, partially resected or recurrent tumors, or for patients with surgical contraindications, or if there is surgical fatigue. The latter can be a major issue in patients with VHL disease (10, 14, 49, 50). However, chances are high that radiosurgery will not influence tumor volume.

The use of high dose intravenous corticosteroids has been described in several case reports, and often because optic neuritis was suspected at initial presentation. Subjective improvement of vision has been described by patients; however, we found no reports on objective improvement as measured by technical examination (12, 16, 35). The administration of corticosteroids seems reasonable for temporary relief of vasogenic edema surrounding hemangioblastomas.

For non-optic nerve VHL related hemangioblastomas, surgical resection remains the gold standard. Pharmacological trials have recently been focusing on the inhibition of Hypoxia Response Elements and their respective receptors, especially vascular endothelial growth factor and receptor (VEGF, VEGFR). There have been pharmacological triumphs in the treatment of VHL clear cell carcinomas, for example with the HIF2α inhibitor belzutifan. Belzutifan also seems to be efficacious for hemangioblastoma tumor control; however, intratumoral hemorrhages have been described, necessitating the development of other therapies. The effect on optic nerve hemangioblastomas has not been investigated and this will remain challenging given their rarity.

## 5 Conclusion

If hemangioblastomas of the optic nerve and chiasm are diagnosed, we suggest annual routine follow-up as long as patients do not develop vision deficiency. If tumors grow fast and/or patients develop vision impairment we recommend resection, because impairment is irreversible, and resection of large tumors carries a higher risk of further vision decline. If the patient is already blind, we recommend surgical resection if MRI shows imminent edema or tumor spread to the chiasm or contralateral optic nerve. Edema can be present before vision impairment becomes clinically evident. If the patient is almost blind, we recommend waiting until full blindness (because vision sparing surgery is unlikely at this stage), unless the contralateral eye is threatened by spreading edema.

## Data availability statement

All relevant data is contained within the article: the original contributions presented in the study are included in the article/supplementary material, further inquiries can be directed to the corresponding author/s.

## Ethics statement

The studies involving humans were approved by Universitair Ziekenhuis Brussel ethics committee. The studies were conducted in accordance with the local legislation and institutional requirements. Written informed consent for participation was not required from the participants or the participants’ legal guardians/next of kin in accordance with the national legislation and institutional requirements.

## Author contributions

EV: Conceptualization, Data curation, Formal analysis, Investigation, Methodology, Visualization, Writing – original draft, Writing – review & editing. J-HK: Conceptualization, Data curation, Formal analysis, Investigation, Writing – original draft. MK: Conceptualization, Data curation, Formal analysis, Investigation, Writing – original draft. CS: Conceptualization, Data curation, Formal analysis, Investigation, Writing – original draft. RK: Data curation, Formal analysis, Writing – original draft, Conceptualization. SR: Data curation, Formal analysis, Investigation, Writing – original draft, Conceptualization. A-MV: Data curation, Formal analysis, Investigation, Writing – original draft, Conceptualization. CA: Writing – original draft, Conceptualization, Methodology. SG: Conceptualization, Data curation, Formal analysis, Investigation, Methodology, Supervision, Validation, Visualization, Writing – original draft, Writing – review & editing.
